# Factors associated with initial AstraZeneca vaccine knowledge, attitudes, and uptake among hospital nurses: A cross-sectional study in Ghana’s Upper East region

**DOI:** 10.1371/journal.pgph.0002674

**Published:** 2024-02-01

**Authors:** Simon Ferguson Atongu, Gifty Apiung Aninanya, Natasha Howard

**Affiliations:** 1 Department of Advanced Nursing Practice, School of Nursing and Midwifery, University for Development Studies, Tamale, Ghana; 2 Department of Health Services Policy, Planning, Management and Economics, School of Public Health, University for Development Studies, Tamale, Ghana; 3 Saw Swee Hock School of Public Health, National University of Singapore and National University Health System, Singapore, Singapore; 4 Department of Global Health and Development, London School of Hygiene and Tropical Medicine, London, United Kingdom; University of Michigan, UNITED STATES

## Abstract

The COVID-19 pandemic has caused over 171,657 confirmed cases and 1,462 deaths in Ghana, particularly among frontline health-workers involved in pandemic response. Prevention measures in Ghana include AstraZeneca ‘Covishield’ vaccination of health-workers, but research on factors affecting uptake of COVID-19 vaccines in Ghana were initially limited. Therefore, this study aimed to analyse knowledge, attitudes, and initial uptake of Covishield among nurses in the War Memorial Hospital in Navrongo, Upper East Region. We conducted a cross-sectional survey of 128 district hospital nurses using simple random sampling. We first calculated descriptive statistics and two composite variables summing either participant knowledge or attitude variables, with a threshold over 50% considered ‘sufficient’ or ‘positive’ respectively. We then analysed associations between demographic variables and Covishield knowledge, attitudes, or uptake using binomial logistic regression with a 95% confidence interval and p-value of <0.05 considered significant. All participants had heard of Covishield vaccine, with attitudes toward it generally positive (53%), and uptake high (72%). Reasons cited by the 28% unvaccinated included absence during vaccination, having already been infected with COVID-19, lack of trust in vaccine safety/efficacy, or pregnancy/breastfeeding. Education, residence, and family type were the only demographic factors significantly associated with nurses’ knowledge about, attitudes towards, or uptake of COVID-19 vaccination. While ‘positive’ attitude was significantly associated with higher odds of being vaccinated (AOR 4.75; 95%CI 1.59–14.1), ‘sufficient’ knowledge was not (AOR 1.33; 95%CI 0.53–3.32). This is the first study in Ghana’s resource-constrained Upper East region to examine health-worker perceptions of a novel vaccine and showed nurses’ knowledge, attitudes, and uptake of COVID-19 vaccination were good overall. Further research is needed to determine how best to address hesitancy and understand findings that attitudes appeared more relevant than knowledge for uptake. Findings are relevant for local health authorities in improving vaccine availability and strengthening emergency risk communication and management of adverse events following immunisation.

## Introduction

Coronavirus disease 2019 (COVID-19), caused by severe acute respiratory syndrome coronavirus-2 (SARS-CoV-2) spread to more than 231 countries and territories [1, 2]. In Africa, COVID-19 was initially confirmed in Egypt on 14 February 2020, and spread to all 54 sovereign African countries, with a total of 12.8 million COVID-19 cases and 258,806 deaths as of 7 July 2023 [3]. This represents approximately 1.9% of cases and 3.8% of deaths reported globally [4]. Ghana reported its first two COVID-19 cases on 12 March 2020 among travellers, with 171,657 confirmed cases and 1,462 deaths by 7 July 2023 [3, 5]. Total numbers of COVID-19 cases and deaths in the Africa region may be somewhat higher, e.g. due to testing/recording limitations, though overall COVID-19 appears to have had less impact in Africa than in other regions such as Europe [4].

On 24 February 2021, Ghana became the first country to receive COVAX vaccines, with a first batch of 600,000 doses of AstraZeneca ‘Covishield’ vaccine, produced by the Serum Institute of India, arriving as part of efforts to improve COVID-19 vaccine equity globally. Covishield contains genetic code for the SARS-CoV-2 virus spike protein inserted into a harmless ‘carrier’ adenovirus [6]. The Ghanaian government commenced mass vaccination on 2 March 2021, almost a year after the first cases were detected, in 43 high-incidence districts in Greater Accra, Ashanti, and Central regions [7]. Since COVID-19 vaccination began, some Ghanaians reported concerns about whether to be vaccinated [8–11]. Despite direct knowledge of the impact of COVID-19, even frontline health-workers expressed COVID-19 vaccine hesitancy [8]. This has several implications, including more health-worker infections and deaths [8, 10, 12], absences, and potential transmission to relatives and clients [9].

Studies of health-worker uptake of COVID-19 vaccines have provided mixed findings globally. Estimated pooled COVID-19 vaccine acceptance in a systematic review of studies in the Africa region found 48% for health-workers and 34% for healthcare students [3]. A cross-sectional study by Alvarado-Socarras et al, on physician perceptions of COVID-19 vaccination in Colombia, showed 77% acceptance [13], while Saied & Kabbash found COVID‐19 vaccine acceptance was only 35% among Egyptian medical students [24]. An online survey of COVID-19 vaccines among health-workers in Saudi Arabia indicated knowledge improved their acceptance of vaccination [12]. Notably, baseline vaccination acceptance and public confidence in vaccination is weak in many countries. In Ghana, a community-based cross-sectional study in Northern, Ashanti and Western North regions found under 42% COVID-19 vaccine acceptability, with 55% having limited knowledge of the vaccine [14]. As of 1 April 2023, COVID-19 vaccine coverage nationally was approximately 66.9%, indicating improvement though more is needed [15]. Studies on Covishield uptake among Ghanaian health-workers are sparse and most such research predated vaccination rollout. Agyekum et al found most health-workers expressed COVID-19 vaccine hesitancy due to safety concerns [8]. An online survey of 1,605 frontline health-workers in 16 regions, prior to COVID-19 vaccine introduction, showed 48% would participate in a COVID-19 vaccine trial if given the opportunity and 70% would accept COVID-19 vaccination [10]. However, another online survey of 234 health-workers showed approximately 39% would accept COVID-19 vaccination, with factors such as sex, cadre, relatives diagnosed with COVID-19, and adverse effects identified as the main reasons for declining vaccination [8, 16]. An online study of 108 clinical radiographers found over half (59%) were willing to be vaccinated, primarily to reduce transmission and lower mortality (55%), while concerns about efficacy and side effects (57%), and fears about racial effects (9%) and fertility (5%) were some reasons for hesitancy [9].

Many of these studies occurred before vaccines were available in Ghana and might not have reflected actual behaviours, nor did they consider Covishield vaccine specifically. Moreover, no studies were conducted among health-workers in Ghana’s Upper East region, which is considerably poorer than most other regions. Additionally, facility reports indicated more than 100 health-workers in Ghana had been infected with COVID-19 and at least 10 had died at the time of our study [17, 18].

We thus aimed to analyse associations of demographic variables and reported COVID-19 knowledge, attitudes, and experiences with Covishield vaccination among nurses in the War Memorial Hospital in Navrongo, Upper East Region. Objectives were to: (i) identify nurses’ knowledge, attitudes, and experiences about COVID-19 and Covishield; and (ii) analyse associations with Covishield vaccine uptake among nurses.

## Methods

### Study design and setting

We chose a cross-sectional survey design to quantify and compare reported knowledge, attitudes, and behaviours of nurses in the War Memorial Hospital (WMH) in Navrongo, Kassena-Nankana East Municipality (KNEM), Upper East Region, from 30 June to 15 August 2021. WMH district hospital was one of the facilities in Ghana hardest hit by COVID-19 when we developed our study, with 15 staff infections and the death of a nurse [18]. Mass staff testing and hospital fumigation were initiated to reduce SARS-CoV-2 spread, health-workers in Navrongo municipality were vaccinated on 25–29 March with two additional days for mop-up, and all residents asked to follow safe management protocols [18].

### Population and sampling

We selected WMH purposively, due to its large sample of health-workers with COVID-19 experience. We selected nurses as they had the most daily exposure to patients and thus to COVID-19, and thus included the eight of its 14 departments that were nurse-managed, i.e. Male ward; Female ward; Neonatal Intensive Care Unit (NICU); Emergency ward; Paediatric ward; Outpatient Department (OPD); Eye Clinic, Ear, Nose and Throat (ENT) Clinic; and Psychiatry and Neurological Unit. We included all 128 nurses in these departments of any cadre or sex who were available during data collection and provided informed consent (i.e. of 150 employed, 19 were absent and 3 did not provide consent but did not differ in gender or cadre from those who did).

### Data collection

The survey instrument, adapted from related literature [8, 19], was pretested in another facility to ensure feasibility. It comprised five sections: (1) socio-demographic characteristics, i.e. age, sex, education level, family type, ethnicity, and place of residence; (2) COVID-19 knowledge and experience; (3) Covishield knowledge; (4) attitudes towards Covishield; and (5) Covishield uptake.

Three trained data collectors met nurses in WMH, described the study and consent process, and answered any questions. Those who agreed to participate signed a consent form, which was stored in a locked cabinet separately from questionnaires. All questionnaires were administered in-person in WMH, with daily checks to ensure completion (i.e. over 95% of questions completed overall). Confidentiality and anonymity were ensured by interviewing nurses in a private room, not including names or identifying information on questionnaires, and assigning each an identification code.

### Study variables

Independent variables were age (i.e. 21–30, 31–40, 41–50), sex (i.e. male, female), marital status (i.e. unmarried, married), family type (i.e. nuclear, extended), residence (i.e. rural, urban), indigeneity (i.e. non-indigenous, indigenous), education level (i.e. 2-year certificate, 3-year diploma, 4-year degree), and cadre (i.e. Registered General Nurse, Registered Nurse Assistant Clinical, Specialist Nurse). For regression analysis, we recoded age (i.e. 21–30, 31–50), ethnicity (i.e. indigenous/non-indigenous), cadre (i.e. Registered General Nurse, Registered Nurse Assistant Clinical/Specialist Nurse) as bivariate variables due to small cell sizes.

Our primary dependent variable was Covishield uptake. Our secondary dependent variables were two binary knowledge and attitudes variables. To obtain our ‘knowledge’ variable, we summed responses for each knowledge question into a composite variable, which we converted to binary using a 50% threshold with less than 50% categorised as ‘insufficient’ and 50% or more categorised as ‘sufficient.’ To obtain our ‘attitudes’ variable, we similarly summed Likert responses for each attitude question and then converted the composite into a binary variable, using a 50% threshold with less than 50% categorised as ‘negative’ and more categorised as ‘positive.’

### Analysis

We used Stata/IC version 16 to clean, code and analyse data. We first summarised data by calculating descriptive statistics, including frequencies and percentages. We then generated the knowledge and attitude variables as described above and analysed associations between demographic variables and knowledge, attitudes, or Covishield uptake variable using binomial logistic regression with age, sex, marital status, family type, residence, ethnicity, and education treated as *a priori* confounders. We used the Hosmer-Lem goodness-of-fit test to assess the model’s fit (p-value = 0.017). We used a confidence interval of 95% with p-value less than 0.05 treated as significant.

### Ethics

We obtained ethics approval from the Kwame Nkrumah University of Science and Technology Ethics Review Board (CHRPE/AP/224/21) before study commencement. Administrative approval was given by the Upper East Regional Health Directorate and the War Memorial Hospital.

## Results

### Demographic characteristics

Table 1 provides demographic characteristics of 128 participating nurses. Most were aged 21–30 (59%), approximately half were women (55%) or married (52%) and none reported being divorced. Approximately half (53%) lived in extended rather than nuclear families and most (69%) lived in urban areas. Of 60 (47%) identifying as indigenous, most (30%) identified as Kassena, 11% as Nankana, and 6% as Builsa. Most held diplomas (48%) and most were Registered General Nurses (64%).

**Table 1:** Demographic characteristics, by Covishield vaccination status, of 128 participating War Memorial Hospital nurses.

Variables	Unvaccinated, n = 36 (%)	Vaccinated, n = 92 (%)	Total, n = 128 (%)
**Age**			
21–30	22 (61)	54 (59)	76 (59)
31–40	11 (31)	36 (39)	47 (37)
41–50	3 (8)	2 (2)	5 (4)
**Sex**			
Male	11 (31)	46 (50)	57 (45)
Female	25 (69)	46 (50)	71 (55)
**Marital status**			
Unmarried	12 (33)	49 (53)	61 (48)
Married	24 (67)	43 (47)	67 (52)
**Family type**			
Extended	25 (69)	43 (47)	68 (53)
Nuclear	11 (31)	49 (53)	60 (47)
**Residence**			
Rural	13 (36)	26 (29)	39 (30)
Urban	23 (64)	66 (71)	89 (70)
**Ethnicity**			
Kassena	14 (39)	24 (26)	38 (30)
Nankana	6 (17)	8 (9)	14 (11)
Builsa	2 (6)	6 (7)	8 (6)
Non-indigenous	14 (39)	54 (59)	68 (53)
**Education (highest achieved)**			
Certificate (2-year tertiary)	16 (44)	19 (21)	35 (27)
Diploma (3-year tertiary)	11 (31)	51 (55)	62 (48)
Bachelor’s degree (4-year tertiary)	9 (25)	22 (24)	31 (24)
**Professional cadre**			
Registered General Nurse	15 (42)	67 (73)	82 (64)
Registered Nurse Assistant Clinical	16 (44)	23 (25)	39 (31)
Specialist Nurse	5 (14)	2 (2)	7 (5)

### Knowledge, attitudes, and experiences related to COVID-19 and Covishield

Table 2 provides frequencies and percentages. Among knowledge variables, all participants (100%) had heard of COVID-19 and the ‘AstraZeneca vaccine’, most initially through television (70%), followed by 27 (20%) through internet/social media, 7 (6%) through radio, 3 (2%) through friends, and only one through relatives (1%). Most (80; 66%) also knew of other COVID-19 vaccines, with 48 (38%) identifying the Pfizer-BioNTech vaccine, 32 (25%) the Janssen vaccine, 28 (22%) the Moderna vaccine, and 27 (21%) the Sputnik V vaccine. Many (71; 56%) reported not knowing about Covishield effectiveness, while 45 (36%) said it was effective or very effective. Most (56%) thought Covishield was not readily available for nurses, while 30% did not know whether it was available. Among those thinking it was not available, most (60; 47%) said the government had not procured enough doses, while 9 (7%) said they did not know why, and 3 (2%) said because senior officials were vaccinated first so there were not enough doses for nurses.

**Table 2:** COVID-19 and vaccine-related knowledge, attitudes, and experiences, comparing 92 vaccinated to 36 unvaccinated War Memorial Hospital nurses.

Knowledge of COVID-19 or Covishield	Unvaccinated, n = 36 (%)	Vaccinated, n = 92 (%)	Total, n = 128 (%)
Heard of COVID-19^1^	36 (100)	92 (100)	128 (100)
Heard of AstraZeneca vaccine (i.e. Covishield)^1^	36 (100)	92 (100)	128 (100)
Heard of other COVID-19 vaccines^1^	21 (58)	59 (64)	80 (63)
*Pfizer-BioNTech*	*11 (31)*	*37 (40)*	*48 (38)*
*Moderna*	*6 (17)*	*22 (24)*	*28 (22)*
*Johnson & Johnson’s Janssen*	*4 (11)*	*27 (29)*	*31 (24)*
*Sputnik*	*5 (14)*	*23 (25)*	*28 (22)*
Source of information about Covishield			n = 128 (100)
*TV*	*26 (72)*	*64 (70)*	*90 (70)*
*Internet/social media*	*6 (17)*	*21 (23)*	*27 (21)*
*Radio*	*2 (6)*	*5 (5)*	*7 (6)*
*Friends*	*1 (3)*	*2 (2)*	*3 (2)*
*Relatives*	*1 (3)*	*0 (0)*	*1 (1)*
How effective is Covishield?^1^			n = 128 (100)
*Don’t know*	*28 (78)*	*50 (54)*	*78 (61)*
*Minimally effective*	*3 (8)*	*2 (2)*	*5 (4)*
*Effective*	*5 (14)*	*39 (42)*	*44 (34)*
*Very effective*	*0 (0)*	*1 (1)*	*1 (1)*
Vaccine is readily available for nurses			n = 128 (100)
*No*	*21 (58)*	*51 (55)*	*72 (56)*
*Yes*	*4 (11)*	*14 (15)*	*18 (14)*
*Don’t know*	*11 (31)*	*27 (29)*	*38 (30)*
If vaccines are not readily available, why not?			n = 72 (100)
*Government could not procure sufficient doses*	*16 (76)*	*45 (88)*	*61 (83)*
*Senior officials were vaccinated before nurses*	*0 (0)*	*3 (6)*	*3 (4)*
*Don’t know*	*5 (24)*	*3 (6)*	*9 (13)*
**Attitudes towards Covishield**			**n = 128 (100)**
Data about Covishield safety are adequate^2^			n = 128 (100)
*Strongly Agree*	*0 (0)*	*0 (0)*	*0 (0)*
*Agree*	*15 (42)*	*44 (48)*	*59 (46)*
*Disagree*	*18 (50)*	*43 (47)*	*61 (48)*
*Strongly disagree*	*3 (8)*	*5 (5)*	*8 (6)*
I encourage family and friends to get vaccinated^2^			n = 128 (100)
*Strongly Agree*	*4 (11)*	*21 (23)*	*25 (20)*
*Agree*	*19 (53)*	*63 (69)*	*82 (64)*
*Disagree*	*11 (31)*	*8 (9)*	*19 (15)*
*Strongly disagree*	*2 (6)*	*0 (0)*	*2 (1)*
It is impossible to reduce COVID-19 incidence without vaccination^2^			n = 128 (100)
*Strongly Agree*	*6 (17)*	*8 (9)*	*14 (11)*
*Agree*	*12 (33)*	*37 (40)*	*49 (38)*
*Disagree*	*11 (31)*	*41 (45)*	*52 (41)*
*Strongly disagree*	*7 (19)*	*6 (7)*	*13 (10)*
If everyone maintained preventive measures, COVID-19 could be eradicated without vaccination^2^			n = 128 (100)
*Strongly Agree*	*11 (31)*	*22 (24)*	*33 (26)*
*Agree*	*13 (36)*	*35 (38)*	*48 (38)*
*Disagree*	*9 (25)*	*29 (32)*	*38 (30)*
*Strongly disagree*	*3 (8)*	*6 (7)*	*9 (7)*
Health-workers should be vaccinated first^2^			n = 128 (100)
*Strongly Agree*	*18 (50)*	*41 (45)*	*59 (46)*
*Agree*	*10 (28)*	*43 (47)*	*53 (41)*
*Disagree*	*4 (11)*	*6 (7)*	*10 (8)*
*Strongly disagree*	*4 (11)*	*2 (2)*	*6 (5)*
The vaccine should be administered free of charge^1^			n = 128 (100)
*Strongly Agree*	*25 (69)*	*68 (74)*	*93 (73)*
*Agree*	*11 (31)*	*22 (24)*	*33 (26)*
*Disagree*	*0 (0)*	*0 (0)*	*0 (0)*
*Strongly disagree*	*0 (0)*	*2 (2)*	*2 (1*.*6)*
Could afford the vaccine if it were not free^1^			n = 128 (100)
*Strongly Agree*	*0 (0)*	*11 (12)*	*11 (9)*
*Agree*	*11 (31)*	*35 (38)*	*46 (36)*
*Disagree*	*14 (39)*	*39 (43)*	*53 (42)*
*Strongly disagree*	*11 (31)*	*7 (8)*	*18 (14)*
I took the vaccine without hesitation^2^			n = 128 (100)
*Strongly Agree*	*0 (0)*	*31 (24)*	*31 (24)*
*Agree*	*0 (0)*	*43 (34)*	*43 (34)*
*Disagree*	*0 (0)*	*15 (12)*	*15 (12)*
*Strongly disagree*	*0 (0)*	*3 (2)*	*3 (2)*
*Not applicable (not vaccinated)*	*36 (28)*	*0 (0)*	*36 (28)*
**Experiences of COVID-19 and Covishield**			**n = 128 (%)**
Had contact with a COVID-19 patient^1^	17 (47)	53 (58)	70 (55)
Relative diagnosed with COVID-19^1^	6 (17)	10 (11)	16 (13)
Relative died of COVID-19^1^	0 (0)	5 (5)	5 (4)
Ever tested for COVID-19^1^	16 (44)	72 (78)	88 (69)
*Positive result*	*5 (31)*	*16 (22)*	*21 (24)*
*Negative result*	*11 (69)*	*56 (78)*	*65 (76)*
Ever vaccinated against COVID-19	*0 (0)*	92 (72)	92 (72)
Experienced any adverse effects	*0 (0)*	n = 71 (77)	n = 71 (77)
*Pain*, *swelling*, *tendency*, *redness*	*0 (0)*	*19 (21)*	*19 (21)*
*Itching at the injection site*	*0 (0)*	*8 (9)*	*8 (9)*
*Fever*, *chills*	*0 (0)*	*32 (35)*	*32 (35)*
*Tiredness*	*0 (0)*	*23 (25)*	*23 (25)*
*Headache*	*0 (0)*	*45 (50)*	*45 (50)*
*Muscle/joint pain*	*0 (0)*	*23 (25)*	*23 (25)*
*Felt unwell*	*0 (0)*	*35 (38)*	*35 (38)*
Received any pre-orientation about the vaccine	*0 (0)*	59 (64)	59 (64)
Found it time-consuming to get vaccinated	*0 (0)*	8 (9)	8 (9)
Adequate safety measures provided during vaccination	*0 (0)*	71 (77)	71 (77)
Got Covid-19 after receiving the first dose	*0 (0)*	1 (1)	1 (1)
Had a second vaccine dose	*0 (0)*	6 (7)	6 (7)
Adverse effects experienced after second dose	*0 (0)*	0 (0)	0 (0)
If not vaccinated, why not?	n = 36 (%)	*0 (0)*	n = 36 (%)
*Not present during vaccination*	*12 (33)*	*0 (0)*	*12 (33)*
*Don’t trust safety of vaccine*	*11 (31)*	*0 (0)*	*11 (31)*
*Was infected with COVID-19 and feared increased severity*	*5 (14)*	*0 (0)*	*5 (14)*
*Don’t trust vaccine effectiveness*	*4 (11)*	*0 (0)*	*4 (11)*
*Breastfeeding and feared vaccine effects on baby*	*2 (6)*	*0 (0)*	*2 (6)*
*Pregnant and feared vaccine effects on unborn child*	*2 (6)*	*0 (0)*	*2 (6)*

NB

^1^ Included in knowledge-experience variable.

^2^ Included in attitudes variable.

Among attitude variables, while 70 (54%) did not consider Covishield safety data sufficient, most (107; 84%) said they would encourage their family and friends to get vaccinated. Approximately half (63; 49%) said vaccination was essential to controlling COVID-19, while most (81; 63%) agreed that if everyone maintained preventive measures the COVID-19 pandemic could be eliminated without vaccination. Most (112; 88%) said health-workers should be vaccinated first and agreed that AstraZeneca vaccination should be administered free-of-charge in Ghana (126; 98%), though 57 (45%) said they could afford vaccination if necessary. Of the 92 nurses vaccinated, 74 (80%) reported taking it without hesitation.

Among experience variables, 70 (55%) had contact with COVID-19 patients and 16 (13%) had relatives diagnosed with or dying (4%) of COVID-19. Most (86; 67%) had been tested for COVID-19 at least once, with most (65; 76%) testing negative. Most (92; 72%) reported getting vaccinated with Covishield. Among those vaccinated, most (71; 77%) experienced some minor adverse effects, primarily headache (45; 49%), while 32 (35%) experienced fever and chills, 23 (25%) experienced tiredness, 23 (25%) experienced muscle/joint pain, 35 (38%) felt unwell, 19 (21%) experienced pain, swelling, or redness at injection sites, and 8 (9%) experienced itching at injection sites. Most reported receiving some pre-vaccination information (59; 64%) and that adequate safety measures were taken during vaccination (71; 77%). Only 8 (9%) considered it time-consuming to get vaccinated and only 1 (1%) reported getting COVID-19 after vaccination. However, only 6 (7%) reported receiving a second dose of Covishield, primarily because the government was not able to procure enough doses. None of these six reported adverse effects with the second dose. Of the 36 (28%) who were not vaccinated, 12 (33%) were not present during vaccination, 11 (31%) did not trust vaccine safety, 5 (14%) had already been infected with COVID-19 and feared they would get more severe disease if vaccinated, 4 (11%) did not think the vaccine was effective against COVID-19, 4 (8%) were breastfeeding or pregnant and feared consequences for their child.

### Associations between demographics and Covishield knowledge, attitudes, or uptake

Knowledge of Covishield was high overall, with 55% scoring above 50% in composite knowledge. Table 3 shows that after adjustment for potential confounders, only education was significantly associated with overall knowledge as bachelor’s degree holders had eight times higher odds of ‘sufficient’ knowledge than nursing certificate holders (AOR 8.01; 95%CI 2.14–29.9).

**Table 3:** Associations between demographic factors and Covishield knowledge, attitudes, or uptake among 128 War Memorial Hospital nurses.

Variables	Knowledge AOR (95% CI)	Attitude AOR (95% CI)	Uptake AOR (95% CI
**Age group**			
21–30	1	1	1
31–50	1.03 (0.68–1.59)	1.12 (0.73–1.72)	1.30 (0.77–2.19)
**Sex**			
Male	1	1	1
Female	2.07 (0.79–5.45)	0.93 (0.37–2.34)	1.02 (0.33–3.19)
**Marital status**			
Unmarried	1	1	1
Married	0.49 (0.18–1.30)	2.04 (0.76–5.41)	0.29 (0.08–1.05)
**Family Type**			
Extended	1	1	1
Nuclear	0.80 (0.35–1.84)	0.41 (0.17–0.99)	5.57 (1.80–17.2)*
**Residence**			
Rural	1	1	1
Urban	1.36 (0.82–2.28)	1.80 (1.13–2.87)*	1.13 (0.42–3.08)
**Indigeneity**			
Not indigenous	1	1	1
Indigenous	1.82 (0.85–3.90)	1.22 (0.56–2.64)	0.45 (0.18–1.13)
**Educational status**			
Certificate	1	1	1
Diploma	1.62 (0.62–4.20)	0.59 (0.22–1.58)	3.89 (1.35–11.2)*
Bachelor’s degree	8.01 (2.14–29.9)*	0.17 (0.05–0.63)*	2.95 (0.78–11.1)
**Professional cadre**			
Reg. General Nurse	1	1	1
Higher cadres	0.95 (0.62–1.46)	1.02 (0.65–1.62)	0.48 (0.29–0.80)
**Knowledge**			
Insufficient	NA	NA	1
Sufficient	NA	NA	1.33 (0.53–3.32)
**Attitude**			
Negative	NA	NA	1
Positive	NA	NA	4.75 (1.59–14.1)*

NB

^1^ Age (i.e. <31, 31+), sex, marriage, family type, residence, indigeneity, and education are treated as a priori confounders.

*Significant at p<0.05 level.

Attitudes towards Covishield were positive overall, with 53% scoring above 50% in composite attitude. Residence and education were significantly associated with Covishield attitude, after adjustment for potential confounders (Table 3). Urban residents had 80% higher odds of positive attitudes than rural residents (AOR 1.80; 95%CI 1.13–2.87). Nurses with bachelor’s degrees had 83% lower odds of a positive attitude towards Covishield than certificate holders (AOR 0.17; 95%CI 0.05–0.63).

Uptake of Covishield was good, with 72% reporting having received the first dose. After adjustment for potential confounders, residence and education were significantly associated with Covishield uptake as was attitude, though knowledge was not (Table 3). Urban residents had over five times higher odds of vaccine uptake than rural residents (AOR 5.57; 95%CI 1.80–17.2). Diploma holders had over three times higher odds of Covishield uptake than certificate holders (AOR 3.89; 95%CI 1.35–11.2). ‘Positive’ attitude was significantly associated with Covishield vaccine uptake, with nurses expressing over 50% positivity having four times higher odds of being vaccinated (AOR 4.75; 95%CI 1.59–14.1). ‘Sufficient’ knowledge was not significantly associated with vaccine uptake (AOR 1.33; 95%CI 0.53–3.32).

## Discussion

### Primary results

To our knowledge, this is the first study analysing health-worker knowledge, attitudes, and uptake of COVID-19 vaccination in Upper East region and certainly in KNEM. This study thus fills a gap in the knowledge of factors influencing COVID-19 vaccine uptake among frontline health-workers in a low-income area of northern Ghana. Previous studies of COVID-19 vaccine knowledge, attitudes, or uptake among health-workers in Ghana occurred before vaccination began [8, 10, 12] and were not specific to nurses, while Agyekum and colleagues demonstrated that interventions to promote COVID-19 vaccination should consider health-worker cadre [8].

### Knowledge

Our results showed sufficient knowledge of COVID-19 and Covishield (i.e. over 50% among 55% of nurses). This is potentially important, given knowledge is considered relevant to health-worker vaccination behaviour and ability to accurately inform service-users, as found in Italy [20] and Saudi Arabia [12]. Our results that only education was significantly associated with knowledge ((i.e. bachelor’s degree holders having eight times higher odds of ‘sufficient’ knowledge than nursing certificate holders (OR 8.01; 95%CI 2.14–29.9)) align with findings in Uganda and Bangladesh that health-workers were significantly more knowledgeable than laypeople about COVID-19 and vaccination [19, 21]. Most of our participants learnt about Covishield through television (70%), which is consistent with Lin et al’s findings of COVID-19 vaccine knowledge among frontline health-workers in Taiwan [22] and Ledda et al’s findings in Italy [20]. Kyei-Arthur found that Ghanaian health-workers whose relatives were diagnosed with COVID-19 had more knowledge about COVID-19 and its consequently, expressed more interest in protecting themselves with COVID-19 vaccines [8].

### Attitudes

Our results showed mixed attitudes towards Covishield (i.e. 53% of nurses). Attitudes are important, as literature indicates that health-worker attitudes affect their intentions to be vaccinated against COVID-19 [20, 23, 24] and that people are more likely to get vaccinated if recommended to do so by a healthcare professional [25]. Our finding that nurses with bachelor’s degrees had 83% lower odds of positive attitudes towards Covishield than certificate holders (AOR 0.17; 95%CI 0.05–0.63) potentially contrasts with the literature on knowledge informing attitudes, e.g. Saied *et al*’s findings that highest COVID-19 vaccine acceptance was among graduate participants (i.e. 47%, 38/81) while highest hesitancy and refusal was among juniors ((i.e. 51% (140/277) and 22% (61/277) respectively)) [24]. However, given some of our questions were specific to Covishield at a time when its safety was being questioned globally, perhaps this is less surprising.

### Uptake

Most of our participants (72%) were vaccinated with Covishield when it was available, aligning with results of another facility-based cross-sectional study, of 424 Ghanaian health-workers, showing 74% acceptance of COVID-19 vaccination [26] and even higher results of 95% and 92% COVID-19 vaccine uptake among Greek [27] and US [28] health-workers respectively. Findings that nurses from nuclear families and with more education had higher odds of getting vaccinated ((i.e. AOR 5.57 (95%CI 1.80–17.2) for nuclear versus extended and AOR 3.89 (95%CI 1.35–11.2) for diploma versus certificate respectively)) may be proxies for income-level or ‘modern’ medicine preferences and more research would be needed to clarify this. Interestingly, our finding that ‘positive’ attitude was significantly associated with Covishield vaccine uptake (i.e. AOR 4.75; 95%CI 1.59–14.1) while ‘sufficient’ knowledge was not (i.e. AOR 1.33; 95%CI 0.53–3.32) indicates attitude is potentially more important than knowledge in determining uptake, though the sample is small and further research is needed.

Among our 36 (26%) unvaccinated nurses, 33% were reportedly absent during vaccination and 11% were concerned about pregnancy and breastfeeding (potentially understandable given lack of clarity at the time about Covishield’s safety during pregnancy despite many countries recommending its use [29], while 20 (56%) expressed general hesitancy related to safety or effectiveness. Though numbers are small, this suggests potential need for better risk communication.

Adverse events following immunisation (AEFI), reported by 77% of our vaccinated participants, included fever, tiredness, headache, muscle/joint pains, and injection site pain/swelling/redness/tenderness, or itching. Another cross-sectional study of Ghanaian health-workers vaccinated against COVID-19 similarly found 81% experienced such reactions, including general weakness (32%), headache (27%), and fever (19% [30]). Similarly, the UK Medicines & Healthcare Products Regulatory Agency states that following vaccination, recipients may experience multiple simultaneous adverse reactions including myalgia/arthralgia, headache, chills, pyrexia, and malaise [31]. Again, clear risk communication that these reactions pass quickly, are rarely dangerous, and what to do if they persist/appear serious seems warranted.

### Practical implications

The main implications of our results relate to vaccine availability, risk communication, and AEFI. First, Ghana’s Health Service and Ministry of Health should work closely with government and international partners to ensure ongoing availability of COVID-19 vaccines and related supplies, ideally provided free-of-charge to health-workers and those unable to pay, as only 45% of nurses indicated they could afford the vaccine on their own. Second, effective risk communication planning and implementation–as required for COVAX support–can address general hesitancy along with concerns specific to pregnant and breastfeeding women and how to address AEFI. Current risk communication approaches should be informed by ongoing operational research. Finally, Ghana Food and Drugs Authority and Joint Covid-19 Vaccine Review Committee should ensure robust AEFI planning and responses to ensure safety and thus strengthen confidence.

### Limitations

Several limitations should be considered. First, this was a small cross-sectional study in one hospital and we did not attempt to be representative of Upper East region or even KNEM due to time and resource constraints. A larger regionally representative sample may have detected additional significant associations. Second, perspectives of other health staff such as doctors, midwives, and allied health professionals were not captured, nor were perspectives of nurses in other facilities. Thus, findings should not be generalised beyond this study population. WMH is the main referral facility within the municipality, and this study does indicate that the knowledge, attitudes, and practices of key health-workers in relation to Covishield vaccine are generally aligned with the available literature.

## Conclusion

This study provides pertinent information on COVID-19 vaccine knowledge, attitudes, and uptake among WMH nurses, showing knowledge and attitudes towards Covishield vaccination were generally positive and uptake was good. Further research is needed to fully interpret findings that attitudes appeared more relevant than knowledge for uptake and how best to address hesitancy. In the interim, strengthening vaccine availability, emergency risk communication, and AEFI information and responses appears useful.
